# Controlling and Tuning the Dispersion Properties of Calcined Kaolinite Particles in Various Organic Solvents via the Modification Method Using Triethoxyvinylsilane and 3-Mercaptopropionic Acid

**DOI:** 10.3390/molecules29174129

**Published:** 2024-08-30

**Authors:** Yongbing Yuan, Xinyu Tang, Junkang Shi, Congshan Zhou, Lijun Li, Honghong Sun, Derek O. Northwood, Kristian E. Waters, Hao Ma

**Affiliations:** 1 Department of Chemistry and Chemical Engineering, Hunan Institute of Science and Technology, Yueyang 414006, China; 2 BGRIMM Technology Group, Metallurgical Research and Design Institute, Beijing 100081, China; 3 Department of Mechanical, Automotive and Materials Engineering, University of Windsor, 401 Sunset Avenue, Windsor, ON N9B 3P4, Canada; 4 Department of Mining and Materials Engineering, McGill University, 3610 University, Montreal, QC H3A 0C5, Canada

**Keywords:** calcined kaolinite, surface affinity modification, vinyltriethoxysilane, 3-mercaptopropionic acid

## Abstract

The surface of calcined kaolinite particles underwent chemical modification using Vinyltriethoxysilane (VTMS) and 3-mercaptopropionic acid (3-MPA). The grafting ratio of VTMS on the calcined kaolinite surface was adjusted by varying its quantity. FT-IR analysis revealed the initial grafting of VTMS onto the kaolinite surface, resulting in the formation of a C=C reactive site on the surface. Subsequently, an olefin click reaction with 3-MPA occurred, leading to the effective grafting of 3-MPA onto the kaolinite surface and the formation of an efficient coating. Thermal analysis indicated that the optimal grafting level was achieved at a modifier content V:K ratio of 0.5. The estimated grafting ratio of the modifier on the kaolinite surface was approximately 40% when V:K was 0.5. Water contact angle and dispersion experiments demonstrated that the surface properties of kaolinite were effectively controlled by this modification approach. At V:K = 0.3, the modified kaolinite particles exhibited good dispersion in both polar and non-polar solvents. In polar solvents, the average particle size of modified kaolinite was below 1100 nm, while in non-polar solvents, it did not exceed 5000 nm. Considering all aspects, a V:K ratio of 0.3 is recommended. Further investigation into the impact of adding 3-MPA on the surface properties of modified kaolinite particles based on V:K = 0.3 revealed that the hydrophilicity of the modified particles could be enhanced. However, it is advised to keep the maximum M:V ratio (3-MPA to kaolinite) at 1.0.

## 1. Introduction

As one of the most important clay resources, kaolin has been widely used in papermaking, coatings, ceramics, rubber, plastics, adsorbents, molecular sieves, and many other industries due to its low price, abundant reserves, good electrical insulation, and a special layered structure consisting of hexagonal crystals [1,2,3,4,5,6]. The literature shows that even at lower loads, adding a sufficient filler to the polymer matrix can effectively improve the physical and chemical properties of polymer materials [7,8,9,10,11,12,13,14,15]. Composite materials can combine the structure and chemical properties of the main polymer matrix and the filler at the same time, so that the composite material has very beneficial physicochemical properties [16,17,18]. Generally speaking, monomer silicon, silica, graphene, nanofibers, etc., are excellent fillers for the preparation of composite materials [19,20], but the high cost, strong hydrophobicity, environmental pollution, and other problems limit the large-scale application of these fillers in industry. Thus, kaolin, as an inexpensive, environmentally friendly, and widely distributed clay, can be used as an alternative to some excellent commodity reinforcement materials.

Kaolinite, as a clay mineral, possesses a highly hydrophilic surface that renders it poorly dispersible in various matrix materials. Therefore, an appropriate surface treatment of kaolinite particles is essential to achieve uniform dispersion in most matrix materials. Over the past few decades, scientists have developed numerous organoclays and organoclay/polymer composites that exhibit outstanding performance. Several effective methods for modifying kaolinite have been proposed, including surface adsorption, ion exchange, polymer and functional molecule grafting, calcination, and dihydroxylation [5,21]. These methodologies enable an efficient dispersion of kaolinite in both polar and non-polar solvents, enhancing its compatibility with polymer matrices. It is evident that chemical grafting facilitates the bonding of grafting molecules to the kaolinite surface via covalent bonds, resulting in a modified kaolinite with superior dispersion and thermodynamic characteristics [21].

In the process of the chemical grafting of layered kaolin, the strong van der Waals force and hydrogen bonding between the adjacent layers of kaolinite make it very difficult to obtain Al-OH in the interlayer space, thus making the grafting reaction difficult [8,10,22]. At present, most researchers use small intercalating agents with high coupling properties such as dimethyl sulfoxide or N-methylformamide to insert them into the layered structure of kaolin first and then carry out subsequent grafting reactions [16,21,23]. However, kaolinite is calcined before the grafting reaction, thereby destroying the strong hydrogen bond between the kaolinite layers to obtain calcined kaolinite. At the same time, a certain amount of Al-OH and Si-OH will still be exposed in the calcined kaolinite particles (which is conducive to the next grafting reaction), which may be due to the exposure of kaolinite to air during the calcination process, so that the kaolinite surface forms an absorbing layer of -OH [24]. Therefore, the use of calcined kaolinite is more conducive to modified grafting and the production of composite materials with superior performance.

Since Plueddemann first proposed the concept of silane coupling agents (SCAs) in 1962 [25], the rich functional group properties of organosilane have provided more methods and ideas for improving the dispersion of clay particles in matrix materials. A variety of SCAs have been synthesized and Si-OH in the silane coupling agent is used to make it very easy to graft on the surface of the clay particles, while the multi-functional group exposed at the other end of SCAs can effectively improve the surface affinity of the clay particles and also introduce more reactive sites to their surfaces, thus bringing more possibilities for the wide application of clay materials [26,27].

At present, most of the research on kaolinite particle modification is focused on modifying the surface of kaolinite to produce a hydrophobic surface to improve the compatibility of kaolinite particles and the polymer matrix, and to improve the hydrophilicity of matrix materials [28,29,30]. The modification method of kaolinite particles results in a uniform dispersion of the modified particles; however, it significantly constrains the selection of solvent systems for composite material preparation. For instance, the use of highly polar solvents like alcohols and amides is common during composite material preparation, yet the modified kaolinite particles are tailored with hydrophobic groups. This hinders the achievement of a homogeneous dispersion of these modified kaolinite particles within the solvent system [31] and is thus unfavorable for the preparation of composite materials. Therefore, it is necessary to develop techniques that can control the uniform dispersion of kaolinite particles in polar and non-polar solvents. In recent years, some researchers have found that TiO_2_ has self-cleaning properties, can be transformed into a hydrophilic material under ultraviolet radiation, and can be automatically reversed to a hydrophobic material under dark conditions. A biphilic material has been developed based on this discovery [32,33]. Modified TiO_2_ particles prepared using this technique can be uniformly dispersed in most organic solvents, but its high cost and low stability has limited its large-scale application in industry. 

In order to achieve a homogeneous dispersion of modified particles in various organic polar and non-polar solvents, it is crucial to investigate strategies for controlling the surface properties of these particles. However, limited research has been dedicated to addressing this challenge and creating modified particles with balanced hydrophilic and hydrophobic attributes. Hence, the primary objective of this study is to develop a straightforward and efficient modification approach to tailor the surface affinity of calcined kaolinite particles, enabling their uniform dispersion in diverse organic solvents.

Here, we present a one-pot method for modifying calcined kaolinite particles using Vinyltriethoxysilane (VTMS) and 3-mercaptopropionic acid (3-MPA). Initially, vinyl groups are grafted onto the particle surface via VTMS, followed by the attachment of carboxyl groups through a ‘click’ reaction between vinyl and sulfhydryl functionalities. The resulting modifier, derived from VTMS and 3-MPA, effectively coats the kaolinite surface, with the exposed silane and carboxyl groups regulating its surface affinity. We discuss the dispersion behavior of these modified kaolinite particles in diverse polar organic solvents. This preliminary work sets the foundation for the future development of organoclay/polymer composites with enhanced properties. Subsequent studies will explore methods to achieve homogeneous organoclay/polymer composites and investigate the mechanical, weather resistance, and stability characteristics of the resulting polymer composites.

## 2. Results and Discussion

### 2.1. FT-IR Spectra of Kaolinite and Modified Kaolinite (Kg)

**Figure 1:**
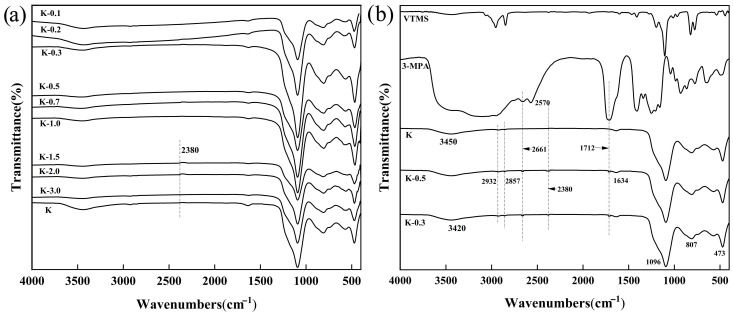
FT-IR spectra of (**a**) K to K-3.0; (**b**) VTMS, 3-MPA, K, K-0.3, and K-0.5.

Figure 1 shows the FT-IR of K, Kg, VTMS, and 3-MPA. In K of Figure 1, the peaks observed at 1096 cm^−1^ and 473 cm^−1^ were attributed to the tensile vibration of Si-O and the telescopic vibration of Si-O-Si, and the peak at 807 cm^−1^ was related to the vibration of Al-O. The peak at 3450 cm^−1^ is the tensile vibration of -OH, and the peak at 1634 cm^−1^ is the telescopic vibration of O-H that absorbs water. Compared to the blank kaolinite K, a series of new peaks appeared in the FT-IR spectrum of modified kaolin such as K-0.5 and K-0.3, and the new peaks of 2932 cm^−1^ and 2857 cm^−1^ were attributed to the tensile vibration peaks of -CH_2_. Through the infrared spectroscopy comparison of 3-MPA, K, K-0.5, and K-0.3, new peaks appeared in the modified kaolinite K-0.5 and K-0.3 at 2661 cm^−1^, 2380 cm^−1^, and 1712 cm^−1^. The absorption peak of 2661 cm^−1^ was identified as the telescopic vibration of S-CH_2_. The peak of 2570 cm^−1^ in the original 3-MPA disappeared in the subsequent modified kaolinite as 2570 cm^−1^ is the telescopic vibration peak of -SH. After the reaction, -SH and C=C were added, resulting in the disappearance of -SH and the -SH telescopic vibration peak at 2570 cm^−1^ and an appearance of a new C-S peak at 2380 cm^−1^. The peak that appeared at 1712 cm^−1^ is the telescopic vibration peak of C=O. The above results show that 3-MPA alone cannot be directly grafted to the kaolin surface, while the C=C exposed by VTMS grafting can be grafted to the kaolinite surface by the olefin click reaction. The -COOH exposed by 3-MPA on the surface of the modified kaolinite will affect the telescopic vibration of -OH, causing the peak of -OH to move to the right to 3420 cm^−1^.

**Figure 2:**
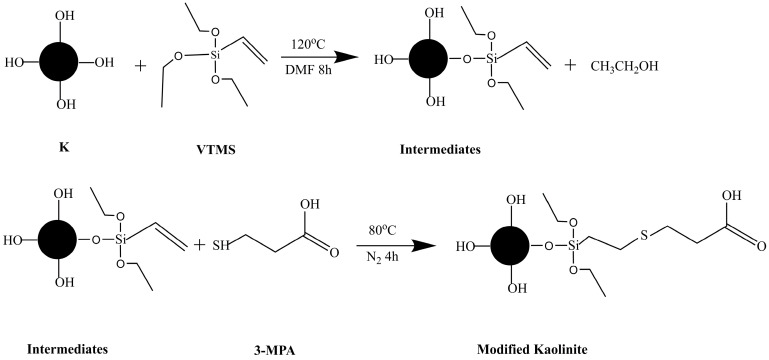
General schematic of surface modification of K particles with VTMS and 3-MPA.

Based on these findings, it is evident that Vinyltriethoxysilane (VTMS) initially undergoes hydrolysis to release -OH groups, followed by a chemical reaction with the surface micro-OH groups of kaolinite to graft VTMS onto its surface, ultimately introducing a new C=C reactive site [34]. The subsequent introduction of 3-mercaptopropionic acid (3-MPA) effectively facilitates the grafting of 3-MPA onto the kaolinite surface via an olefin click reaction, leading to the formation of a surface coating on the kaolinite, as illustrated in Figure 2.

### 2.2. Thermal Analysis

**Figure 3:**
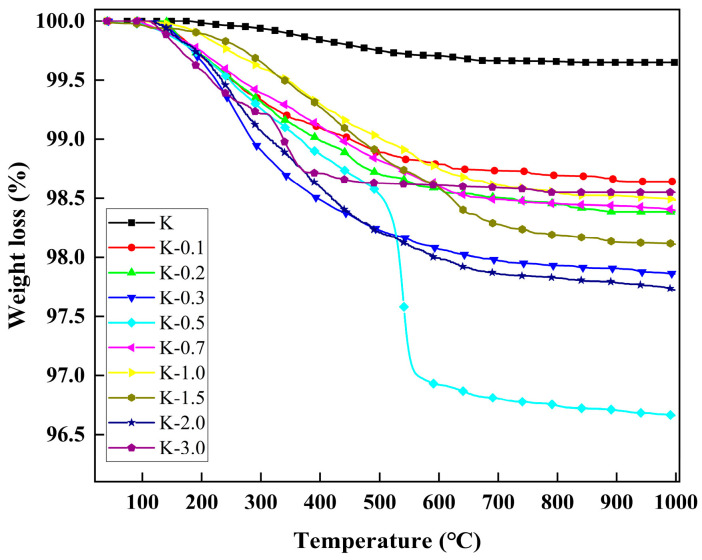
TG curves of K to K-3.0.

**Figure 4:**
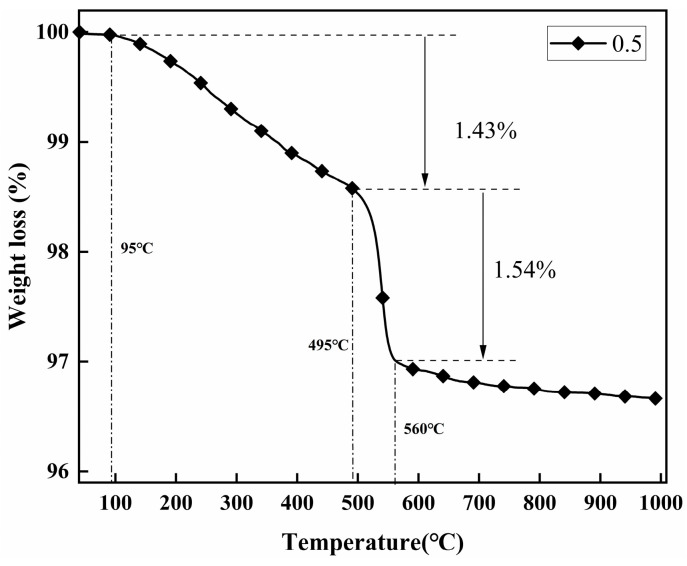
TG curves of K-0.5.

The thermogravimetric variances between kaolinite and different modified kaolinite particles were investigated in the temperature range of 40 °C to 1000 °C, with the results depicted in Figure 3. It is evident that the weight loss exhibited by each modified kaolinite particle surpasses that of pure kaolinite, indicating the successful surface grafting of modifiers onto the kaolinite particles. The highest degree of grafting is achieved at a modifier content ratio of V:K = 0.5. At this ratio, the thermal decomposition behavior is illustrated in Figure 4, where weight loss between 96 °C and 485 °C is attributed to the degradation of water and 3-mercaptopropionic acid within the sample. Subsequently, a rapid weight loss from 485 °C to 577 °C is observed, corresponding to the swift decomposition of grafted VTMS present on the modified kaolin surface.

Thermogravimetric analysis can also quantitatively analyze the grafting ratio of modifiers on the surface of kaolinite particles [24], which can be estimated by the following formula:

(1) $$N_{t}=\frac{2\Delta mk}{M_{H_{2}O}}$$

**Figure 5:**
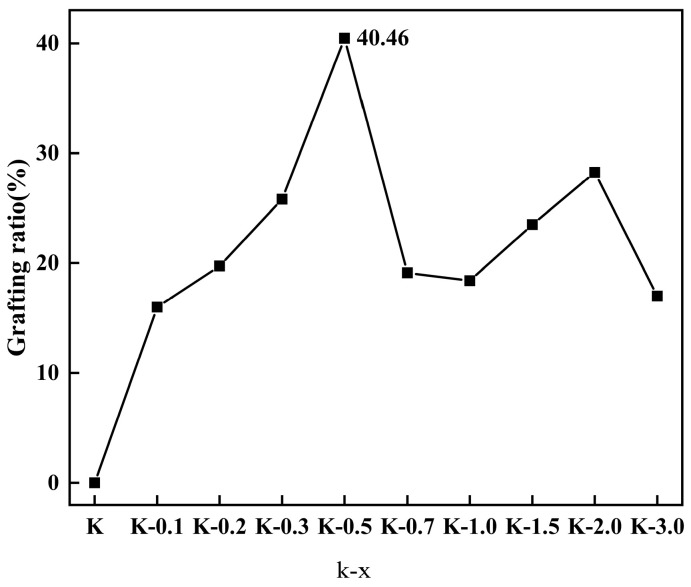
The relationship between the grafting and modified ratios.

N_u_ = N_t_ − N_r_ (2)

(3) $$N_{r}=\frac{2\Delta m_{kg}-N_{u}*M_{H_{2}O}}{2M_{S}}$$

(4) $$S_{C}=10^{6}\frac{N_{r}}{SSA}$$

where N_t_, N_u_, and N_r_ represent the total, unreacted, and reacted hydroxyl groups of raw K particles (mol/g), respectively; Δm_k_ and Δm_kg_ are the weight lost by kaolinite particles and subsequent modified kaolinite particles at 40–1000 °C (mg); MH_2_O and M_S_ are the relative molecular mass of water molecules (18 g/mol) and the average relative molecular mass of grafted modifiers (144 g/mol); S_C_ is the grafting ratio; and SSA is the specific surface area of K. The grafting ratios are shown in Table 1 and Figure 5.

In Table 1, M_1_ and M_2_ represent the initial and final mass of the sample during the thermogravimetric analysis process, respectively. Weight loss (mg) represents the mass loss of the sample during the thermogravimetric analysis process, and weight loss (%) is the weight loss divided by the initial mass of the sample.

Figure 5 illustrates a notable decline in the grafting ratio as the added VTMS amount increases. This phenomenon can be elucidated as follows: at low VTMS dosages, hydrolysis generates silanol species that readily undergo condensation reactions with the hydroxyl groups present on the kaolinite surface. With an elevation in the VTMS dosage, the unreacted hydroxyl groups on the kaolinite surface become occupied by the reacted VTMS molecules, leading to a reduction in the likelihood of subsequent reactions due to steric hindrance [35]. Consequently, the unreacted VTMS, upon hydrolysis, tends to interact with the exposed VTMS-derived silanol species on the kaolinite surface, facilitating the completion of condensation reactions amongst VTMS moieties. Following the initial reaction between a kaolin surface hydroxyl group and a VTMS-derived silicon hydroxyl group, the remaining ethoxy groups on the VTMS molecule can undergo further hydrolysis and condensation with other VTMS units. Through multiple condensations, a significant number of Si-O groups persist on the kaolinite surface. During thermogravimetric analysis, the organic chains decompose and fracture, while the Si-O groups endure. Consequently, only the carbon, sulfur, and hydrogen components of the organic chains undergo thermal decomposition, rather than all the grafted modifier molecules, thereby attenuating the sample’s thermal weight loss rate. Hence, an increased VTMS dosage results in a diminished thermal weight loss rate. Consequently, a low thermogravimetric loss rate may not solely reflect a decrease in the quantity of organic matter grafted onto the kaolinite surface; the contribution of residual Si-O groups must also be acknowledged. This deduction finds support in contact angle analysis results, where an escalating VTMS dosage is accompanied by a continuous increase in the contact angle. Conversely, if the organic matter reaction diminishes with increasing VTMS dosage (e.g., transitioning from K-0.7 to K-3.0), the contact angle would not exhibit a continued rise but rather a decrease.

**Figure 6:**
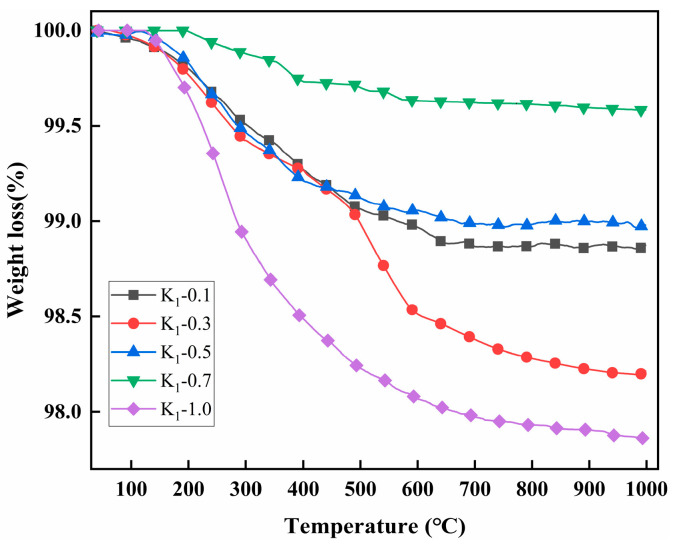
TG curves of K_1_-0.1 to K_1_-1.0.

The impact of varying the subsequent addition ratio of 3-MPA on the thermogravimetric analysis of kaolinite was investigated, with the results illustrated in Figure 6. The findings indicate a lack of discernible correlation between the quantity of 3-MPA introduced and its thermal degradation influence. This observation stems from the primary mode of modification reaction, which predominantly involves grafting onto the surface of kaolinite particles via VTMS. Consequently, when the quantity of VTMS added remains constant, alterations in the grafting effect due to variations in 3-MPA addition are minimal.

### 2.3. Water Contact Angle Analysis

The water contact angle between K and Kg was measured to determine the affinity of modified kaolinite for water, and the results are as follows.

**Figure 7:**
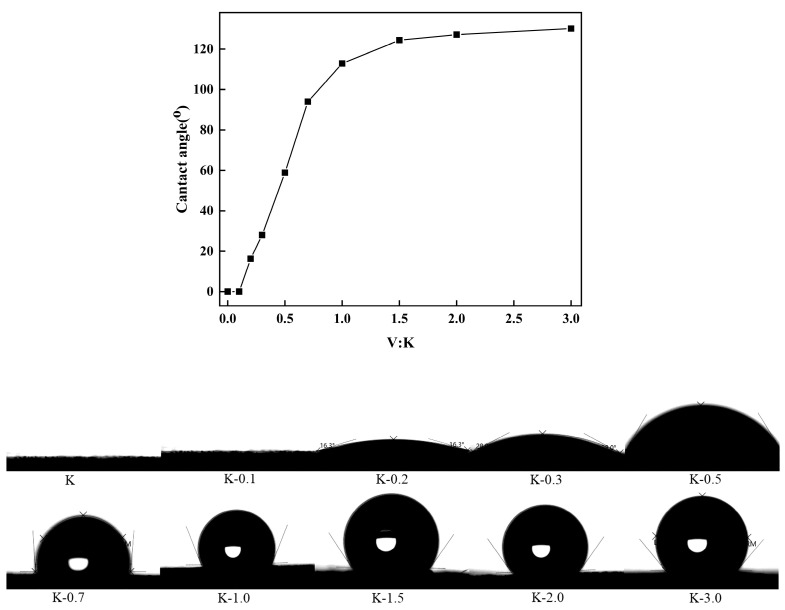
Contact angle of K to K-3.0.

As depicted in Figure 7, the high-speed camera effectively captured the water contact angle between kaolinite and modified kaolin in its pristine state. It is evident that with an increase in VTMS addition, the droplets adhering to the kaolinite surface become more pronounced and rounded, signifying a gradual enhancement in hydrophobicity. Furthermore, the coating on the kaolinite surface becomes more discernible.

The incorporation of VTMS leads to a notable enhancement in the hydrophobicity of kaolin particles, especially within the V:K ratio range of 0.1–1.0, where the modification rapidly boosts the hydrophobic properties of the particles. Conversely, when the V:K ratio exceeds 1.0, the modification effect on the kaolinite diminishes, potentially attributed to a progressive coating of the kaolinite surface by the modifiers.

**Figure 8:**
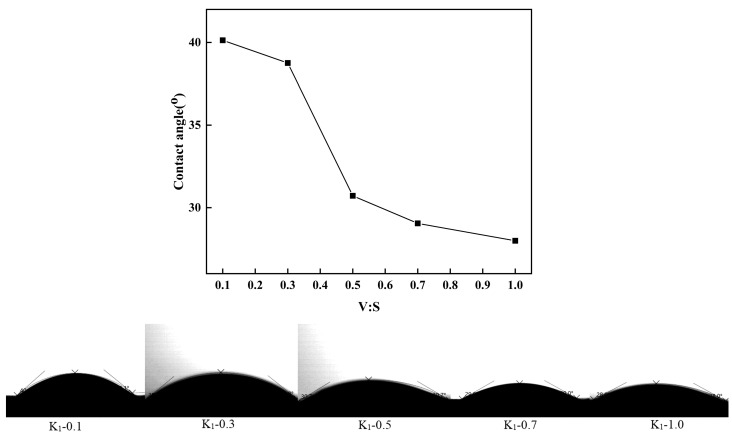
Contact angle of K_1_-0.1 to K_1_-1.0.

The impact of subsequent 3-MPA addition on the contact angle of the kaolinite particles was examined, with the results presented in Figure 8.

The water contact angle exhibits a fluctuating trend in response to the varying amounts of 3-MPA, resulting in an enhancement of hydrophilicity in the modified particles. The -COOH moiety present in 3-MPA plays a crucial role in augmenting the hydrophobic characteristics conferred by VTMS onto the kaolinite particles. Through meticulous adjustments of the K, VTMS, and 3-MPA ratios, it is feasible to achieve the modification of the kaolinite particles into inorganic nanoparticles that simultaneously exhibit hydrophilic and hydrophobic properties.

### 2.4. Dispersion Properties of K_g_ Particles in Various Organic Solvents

#### 2.4.1. K_g_ Dispersibility in Polar Solvents

**Figure 9:**
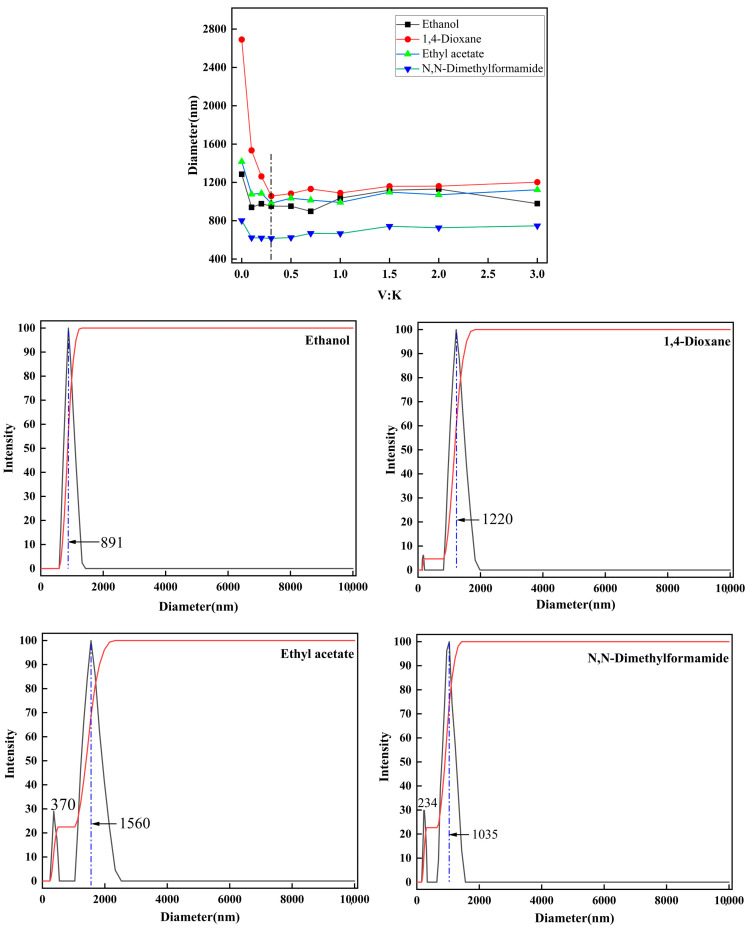
Diameter of ethanol, 1,4-Dioxane, ethyl acetate, N,N-Dimethylformamide, and particle size analysis when the V:K ratio is 0.3.

K and K_g_ were uniformly dispersed into organic solvents using a high-speed homogenizer, and the particle size in various organic solvents was then determined with a particle size analyzer. Its dispersibility in polar solvents is shown in Figure 9.

Figure 9 illustrates that the particle size of the modified kaolinite particles in various polar solvents is significantly smaller compared to that of the kaolinite in polar solvents, indicating that the modification approach facilitates an enhanced dispersion of kaolinite particles in polar solvents. Specifically, when the V:K ratio is 0.3, it is observed that among the four polar solvents examined, the modified kaolinite particles exhibit smaller particle sizes and superior dispersion efficiency. Notably, in ethanol, the peak particle size is 891 nm, with an average size of 952 nm; in dioxane, the peak size is 1220 nm, with an average size of 1057 nm; in ethyl acetate, the peak size is 1560 nm, with an average size of 983 nm; and in DMF, the largest distribution size is 1035 nm, with an average size of 615 nm. Both kaolinite and modified particles demonstrate optimal dispersibility in DMF, warranting its selection for further investigation. However, the particle size distribution of kaolinite particles in polar solvents appears non-uniform, with a small number of kaolinite particles appearing near 170 nm in dioxane, 370 nm in ethyl acetate, and 234 nm in DMF. This phenomenon can be attributed to the disruption of hydrogen bonds on the surface of the modified kaolinite particles during high-speed homogenization, resulting in the presence of free kaolinite particles in the solution system, which are detected as smaller particles with improved dispersion in particle size analysis.

**Figure 10:**
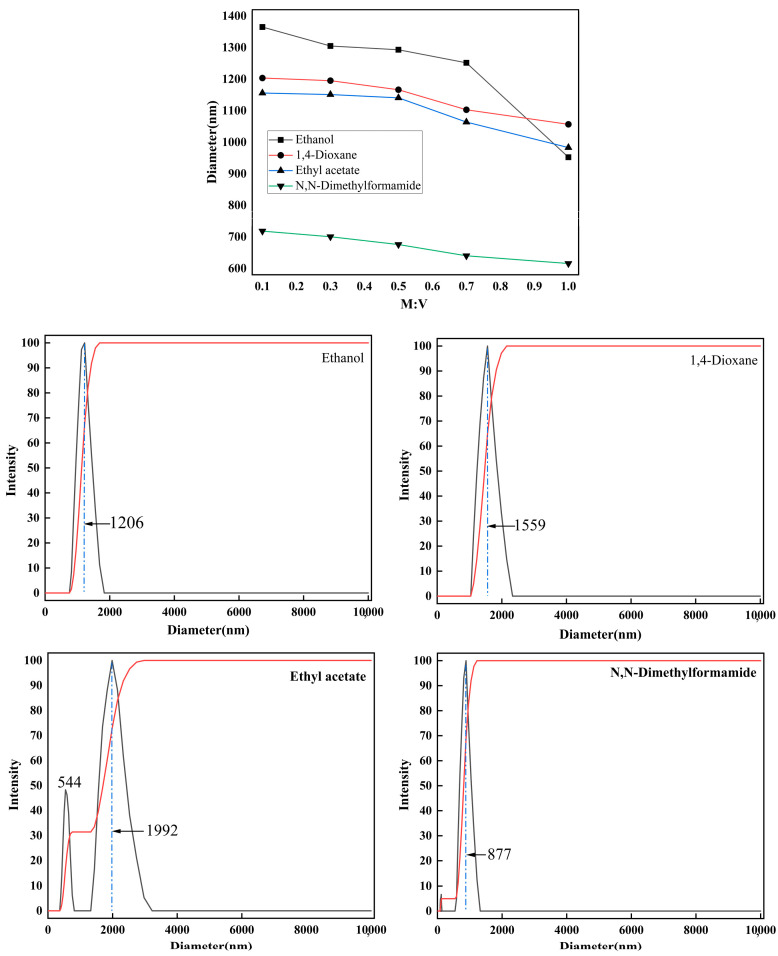
Diameter of ethanol, 1,4-Dioxane, ethyl acetate, and N,N-Dimethylformamide, and particle size analysis when the M:V ratio is 0.1.

The impact of the subsequent addition of 3-MPA on the dispersibility of kaolinite particles in polar solvents was investigated, and the findings are presented in Figure 10.

As depicted in Figure 10, within the polar-solvent environment, the kaolinite particle size diminishes as the quantity of 3-MPA increases, leading to an enhanced dispersion of modified kaolinite. However, it is crucial to note that optimal dispersion is not achieved through maximal 3-MPA addition due to the intrinsic hydrophilicity of kaolinite. The dosage of 3-MPA should not surpass M:V = 1.0. For instance, when the M:V ratio is 0.1, the peak distribution particle size dispersed in ethanol measures 1206 nm, with an average particle size of 1365 nm; in dioxane, the peak distribution size is 1559 nm, with an average size of 1203 nm; ethyl acetate showcases a peak distribution size of 1992 nm and an average size of 1156 nm; and DMF demonstrates the most extensive distribution at 877 nm, with an average size of 718 nm. At V:K = 0.3 (M:V = 1.0), as the quantity of 3-MPA increases, the average particle size of kaolinite particles in polar solvents decreases, underscoring that the simultaneous adjustment of VTMS and 3-MPA addition enables the effective attainment of modified kaolinite particles with desired surface characteristics.

#### 2.4.2. Dispersion of Kg in Non-Polar Solvents

**Figure 11:**
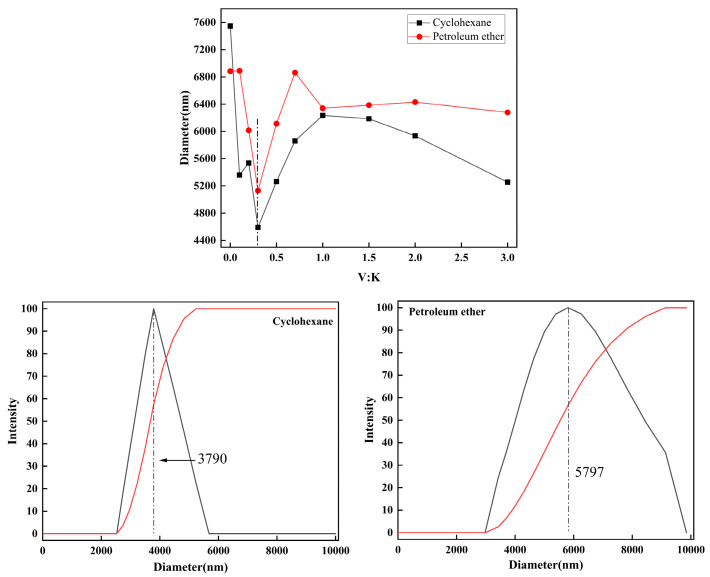
Diameter of cyclohexane, petroleum ether, and particle size analysis when the V:K ratio is 0.3.

As illustrated in Figure 11, in a polar-solvent environment, the size of kaolinite particles decreases with increasing amounts of 3-MPA, resulting in an improved dispersion of the modified kaolinite. It is essential to recognize that maximal dispersion is not achieved by simply adding more 3-MPA, given kaolinite’s inherent hydrophilicity. The quantity of 3-MPA added should not exceed M:V = 1.0. For example, at an M:V ratio of 0.1, the peak distribution particle size dispersed in ethanol measures 1206 nm, with an average size of 1365 nm; in dioxane, the peak distribution size is 1559 nm, with an average of 1203 nm; ethyl acetate exhibits a peak distribution size of 1992 nm and an average size of 1156 nm; and DMF displays the widest distribution at 877 nm, with an average size of 718 nm. With V:K at 0.3 (M:V = 1.0), as the amount of 3-MPA increases, the average particle size of the kaolinite particles in the polar solvents decreases, indicating that the concurrent adjustment of VTMS and 3-MPA addition facilitates the effective production of modified kaolinite particles with desired surface properties.

**Figure 12:**
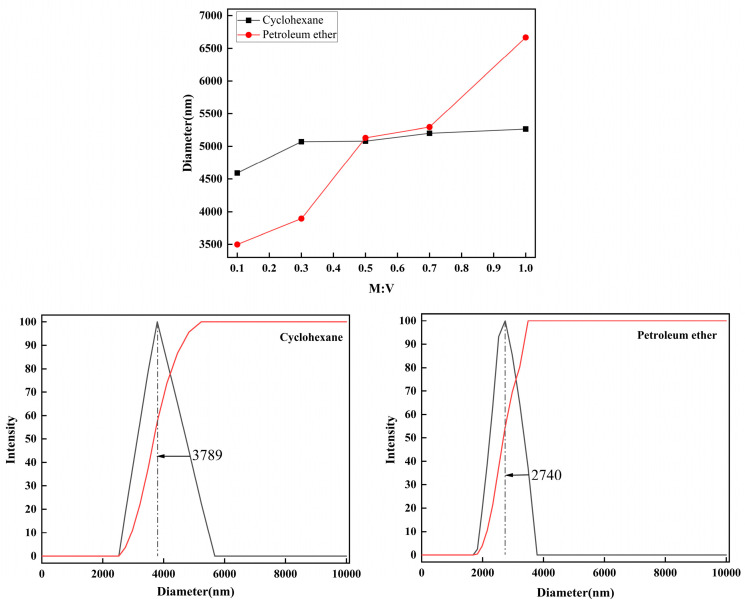
Diameter of cyclohexane, petroleum ether, and particle size analysis when the M:K ratio is 0.1.

The effect of the subsequent addition of 3-MPA on the dispersibility of kaolinite particles in non-polar solvents was investigated, and the results are shown in Figure 12.

In non-polar solvents, the introduction of 3-MPA leads to an increase in the particle size of modified kaolinite particles and a reduction in their hydrophobicity. The dispersibility of modified particles in petroleum ethers is greatly impacted by the quantity of 3-MPA added. For instance, at an M:V ratio of 0.1, the peak particle size in cyclohexane is 3789 nm, with an average size of 4590 nm, while in petroleum ether, the most prevalent particle size is 2740 nm, with an average size of 3498 nm.

### 2.5. Scanning Electron Microscopy

**Figure 13:**
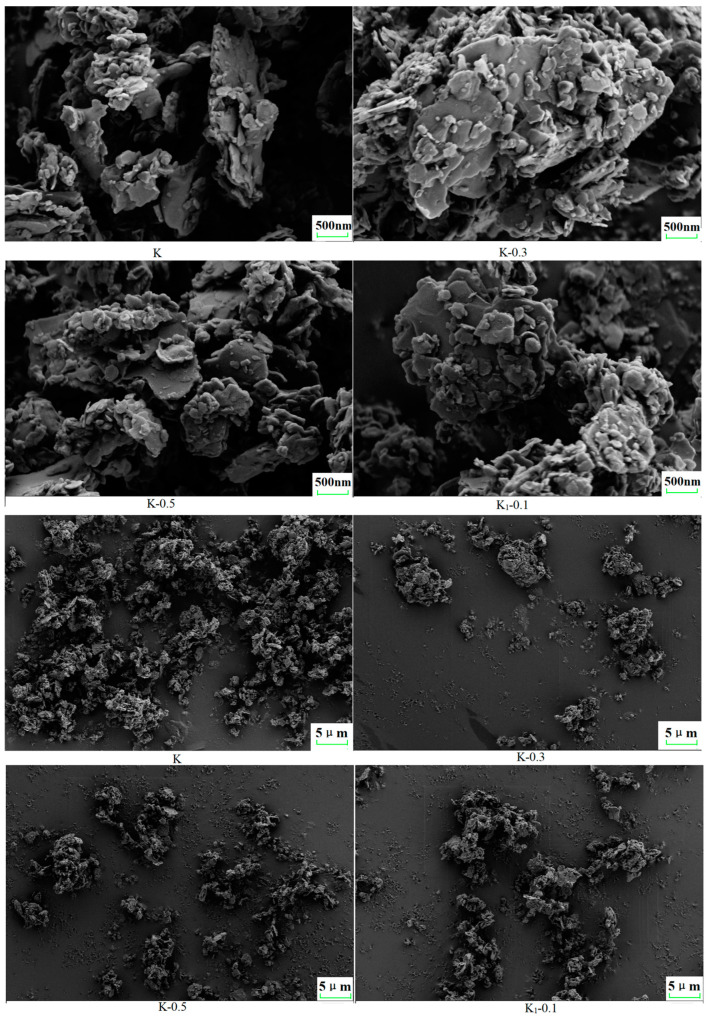
Scanning electron microscopy images of K, K-0.3, K-0.5, and K_1_-0.1 at 500 nm and 5 μm.

Figure 13 displays the scanning electron microscope (SEM) images of K, K-0.3, K-0.5, and K1-0.1. It reveals that the surface of calcined kaolinite (K) primarily exhibits a layered structure composed of crystalline sheets with noticeable voids. Upon the addition of modifiers, the crystalline sheet arrangement within the kaolinite grains remains largely unchanged, albeit with a reduction in the inter-sheet spacing. This phenomenon is attributed to the penetration of the modifier into the interlayer spaces of the kaolinite particles, effectively narrowing the gaps between the sheets. While unmodified K can be smoothly attached to the electrode surface, modified kaolinite particles tend to agglomerate. This observation underscores the substantial enhancement in the surface affinity of kaolinite particles brought about by the modification process.

## 3. Experimental

### 3.1. Materials

The elemental analysis results of calcined kaolinite (K), with 6000 mesh (2.5 μm) and a K specific surface of 12.5 m^2^/g are listed in Table 2.

Kaolinite is ground and dried before each use. N,N-dimethylformamide (DMF) is analytical-grade purity. K and DMF were purchased from the Chinese Rhawn Chemical Reagent Network (Shanghai, China). VTMS (98%) and 3-MPA (99%) were purchased from the Chinese Tansoole platform (Shanghai, China). Ethanol (99.7%), cyclohexane (99.5%), ethyl acetate (99%), dioxane (99%), and petroleum ether, all of analytical-grade purity, were purchased from the China Macklin Chemical Reagent Network (Shanghai, China). 

### 3.2. Preparation of Modified Kaolin Particles by One-Pot Method

In total, 4 g of kaolinite was ground and then dried in a 100 °C oven for 2 h. The resulting sample was dispersed in 100 g of dimethylformamide (DMF) and stirred at room temperature for 15 min. Subsequently, 0.4 g of vinyltrimethoxysilane (VTMS) was added dropwise to the dispersion, followed by stirring at 120 °C for 8 h. The temperature was then reduced to 80 °C, and nitrogen gas was purged for 15 min to eliminate all air from the reaction system. Next, 0.066 g of 3-mercaptopropionic acid (3-MPA) was introduced. Under continuous nitrogen flow (at a rate of 40 mL/min), the mixture was stirred evenly for 4 h. Upon completion of the reaction, the resulting slurry underwent centrifugation and was washed five times with 99.7% ethanol to remove any residual reagents in the modified kaolin. The samples were subsequently dried in an oven at 80 °C for 24 h to yield modified kaolinite K-0.1, with varying amounts of VTMS used (ranging from 0 to 12 g). The quantity of 3-MPA added corresponded to a 1:1 ratio with the amount of VTMS employed.

The products of these reactions were designated as K, K-0.2, K-0.3, K-0.5, K-0.7, K-1.0, K-1.5, K-2.0, and K-3.0. The sample that exhibited optimal V:K dispersion performance was chosen, and subsequent additions of 3-MPA were made at varying ratios of M:V = 0.1, 0.3, 0.5, 0.7, and 1.0. These samples were denoted as K1-0.1, K1-0.3, K1-0.5, K1-0.7, and K1-1.0.

### 3.3. Characterization

FT-IR spectra of the samples were obtained using an AVATAR370 FTIR spectrometer (Richmond Scientific, Bengaluru, Karnataka) with KBr disc technology over the range of 4000 to 400 cm^−1^ at a resolution of 2 cm^−1^. Thermogravimetric analysis was conducted using a NETZSCH STA 449F3-0751-M thermogravimetric analyzer (Netzsch, Selb, Germany), with a heating rate of 20 °C/min from 40 °C to 1000 °C under a nitrogen gas flow of 40 mL/min for protection. Contact angle measurements were taken using a CA100 contact angle analyzer (INNO Precision Instruments, Shanghai, China), with the 10th frame of the captured image uniformly chosen as the contact angle photo. The dispersed particle size of the samples in different polar and non-polar solvents was determined with a NanoBrook 90 plus PALS multi-angle particle size analyzer and high-sensitivity potentiometer (Brookhaven Instruments, Nashua, NH, USA). Prior to each measurement, the sample was thoroughly dispersed in the solvent using a high-speed homogenizer at 25,000 r/min for 1 min to ensure complete scattering. The surface topography of the samples was observed using a ZEISS Sigma300 field emission scanning electron microscope (ZEISS, Jena, Germany).

## 4. Conclusions

The surface of the kaolinite particles was modified with VTMS and 3-MPA to facilitate their uniform dispersion in both polar and non-polar solvents. The surface affinity of the resulting modified kaolinite particles can be controlled by adjusting the ratio of VTMS, 3-MPA, and kaolinite particles during the grafting process. The modifier applied to the Kg particles’ surface consists of hydrophobic silane chains and hydrophilic -COOH groups, enhancing the dispersion of the modified particles in various solvents and mitigating the tendency of the kaolinite particles to agglomerate in non-polar solvents.

This study serves as an initial exploration towards developing organoclay/polymer composites with superior properties. Future investigations will focus on achieving homogeneous organoclay/polymer composites and evaluating their thermal, mechanical, fire retardant, and barrier characteristics. It is important to highlight that the decomposition rate of the modified particles accelerates above 200 °C, warranting further inquiry into how the modified particles enhance the mechanical properties, weather resistance, and stability of polymer composites.

## Figures and Tables

**Table 1:** Grafting ratio of K to K-3.0.

Samples	M_1_ (mg)	M_2_ (mg)	Weight Loss (mg)	Weight (%)	Grafting Ratio (%)
K	20.34	20.26	0.07	0.35	0.00
K-0.1	20.15	19.88	0.27	1.34	16.00
K-0.2	20.81	20.48	0.33	1.60	19.73
K-0.3	20.36	19.92	0.44	2.14	25.81
K-0.5	20.44	19.76	0.68	3.34	40.46
K-0.7	20.29	19.97	0.32	1.59	19.11
K-1.0	20.56	20.25	0.31	1.51	18.39
K-1.5	20.55	20.16	0.40	1.93	23.50
K-2.0	20.91	20.43	0.48	2.28	28.25
K-3.0	20.21	19.93	0.29	1.42	17.00

**Table 2:** Chemical composition of the ore.

Element	Fe_2_O_3_	TiO_2_	CaO	K_2_O	P_2_O_5_	MgO	Na_2_O	Al_2_O_3_	SiO_2_
Con.(%)	0.419	0.678	0.177	0.263	0.382	0.206	0.424	49.48	47.89

## Data Availability

The original contributions presented in the study are included in the article, further inquiries can be directed to the corresponding authors.
